# Strong In-plane Magnetic Anisotropy in Semiconducting Monolayer CoCl_2_

**DOI:** 10.1021/acsnano.5c02175

**Published:** 2025-05-30

**Authors:** Samuel Kerschbaumer, Sebastien Elie Hadjadj, Andrea Aguirre-Baños, Danilo Longo, Andrés Pinar Solé, Oleksandr Stetsovych, Adriana Elizabet Candia, Paula Angulo-Portugal, David Caldevilla, Fadi Choueikani, Martina Corso, David Serrate, Jorge Lobo-Checa, Pavel Jelínek, Maxim Ilyn, Celia Rogero

**Affiliations:** † 202635 Centro de Física de Materiales (CSIC/UPV-EHU), 20018 Donostia-San Sebastián, Spain; ‡ 138823 CIC nanoGUNE-BRTA, 20018 Donostia-San Sebastian, Spain; § 86889 FZU – Institute of Physics of the Czech Academy of Sciences, Cukrovarnická 10, Prague 6 CZ 16200, Czech Republic; ∥ Laboratorio de Microscopias Avanzadas (LMA), Universidad de Zaragoza, E-50018 Zaragoza, Spain; ⊥ Synchrotron SOLEIL, 91190 Saint-Aubin, France; # Instituto de Nanociencia y Materiales de Aragón (INMA), CSIC-Universidad de Zaragoza, 50009 Zaragoza, Spain; ∇ Departamento de Física de la Materia Condensada, Universidad de Zaragoza, E-50009 Zaragoza, Spain

**Keywords:** 2D ferromagnetic materials, van der Waals semiconductors, monolayer CoCl_2_, in-plane magnetism, magnetic thin films, transition-metal dihalides

## Abstract

Transition-metal dihalides (TMDH) are emerging as a highly promising class of 2D magnetic materials due to their simplicity, stability, and compatibility with nanofabrication techniques. In this work, we explore the structural, electronic, and magnetic properties of monolayer CoCl_2_ grown epitaxially on Au(111) using a multitechnique approach. Our results reveal that epitaxial CoCl_2_ exhibits ferromagnetic order below 24 K with strong in-plane magnetic anisotropy, setting it apart from other TMDH materials. Additionally, we identify in-gap states arising from the CoCl_2_–Au­(111) interface, which provide insights into its electronic behavior. These findings position CoCl_2_ as a versatile 2D material for spintronic applications and nanoscale devices, bridging the gap between fundamental research and real-world technological solutions.

## Graphical abstract

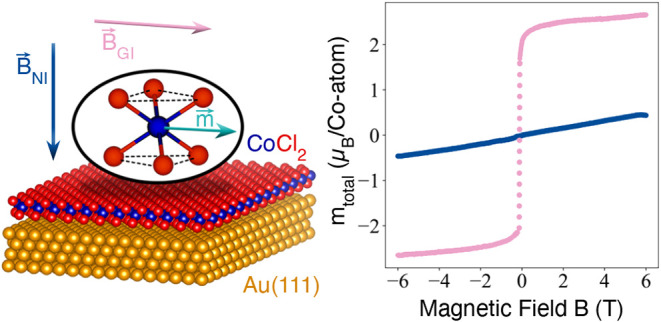

The ever-growing interest to substitute silicon with more economic and efficient nanomaterials in the market for next-generation electronics has led to the discovery and characterization of many new 2D materials.^1–5^ Magnetic 2D materials have gained increasing interest, due to their potential applications as ultrathin barriers for spin-polarized tunneling in devices or for fabricating superconductor-ferromagnet heterostructures.^6–9^ Numerous theoretical calculations predict magnetic order in various 2D materials,^10–14^ but only a few have been experimentally confirmed at the single-layer limit.^4,15–19^ Since the first experimental demonstration of CrI_3_ ^4^ and Cr_2_Ge_2_Te_6_,^15^ other materials have been investigated,^8,14^ including transition-metal dichalcogenides (TMDC) and transition-metal dihalides (TMDH).^20,21^ These 2D magnets are considered valuable building blocks for inducing proximity magnetization effects in van der Waals heterostructures. A common technique to obtain monolayer samples is by exfoliating bulk single crystals. It has been shown that materials in the TMDH family can be synthesized by sublimating stoichiometric powder onto single crystal substrates maintained at relatively low temperatures (around 100 °C), a key advantage for compatibility with a wide range of nanofabrication techniques.^22,23^

Monolayers of TMDH compounds have a 1T crystal structure where two halogen layers are enclosing a triangular lattice of transition-metal atoms, creating an octahedral coordination^10^ (Figure S1). The crystal field from the halogens splits the d-orbitals, thereby influencing the magnetic behavior. The Goodenough–Kanamori–Anderson (GKA) rules^24^ describe superexchange interactions in these wide-bandgap semiconductors, where indirect hopping between metal atoms determines magnetic ordering with antiferromagnetic (AFM) or ferromagnetic (FM) arrangement, depending on the orbital occupation. In recent years, collinear and noncollinear FM ordering with relatively weak magnetic anisotropy have been reported in monolayers of Ni and Fe TMDH compounds grown on Au(111).^16,17,19^ In this work, we present a comprehensive multitechnique investigation that provides clear evidence of the structural, electronic, chemical, and magnetic properties of thin films of the semiconducting van der Waals material CoCl_2_ grown on Au(111). This material turns out to exhibit a very strong magnetic anisotropy, which contrasts with the properties of Ni and Fe compounds. Our study offers a detailed exploration of how CoCl_2_ behaves at the monolayer level, revealing its potential in advanced nanoscale applications and offering insights into the underlying physics.

## Results

Analogous to FeCl_2_ and NiCl_2_,^19^ CoCl_2_ grows in a layer-by-layer fashion when deposited onto Au(111). Figure 1a shows the growth of CoCl_2_ in scanning tunneling microscopy (STM) in the submonolayer (sub-ML) regime. The large-scale topography shows three regions: the herringbone reconstruction of clean Au(111), large first-layer islands, and small second-layer islands growing on top. The first-layer islands have the ability to seamlessly overgrow step edges as a continuous film (see also Figure S5), indicating weak interaction with the substrate and a high degree of flexibility and stability arising from strong bonds within the layer. Another indicator is the preservation of the herringbone reconstruction, which remains unaffected beneath the partially transparent material. Notably, the second layer is observed before the gold surface is fully covered. This implies that thermally driven motion on top of the first layer is reduced compared with the Au(111) substrate.

**Figure 1:**
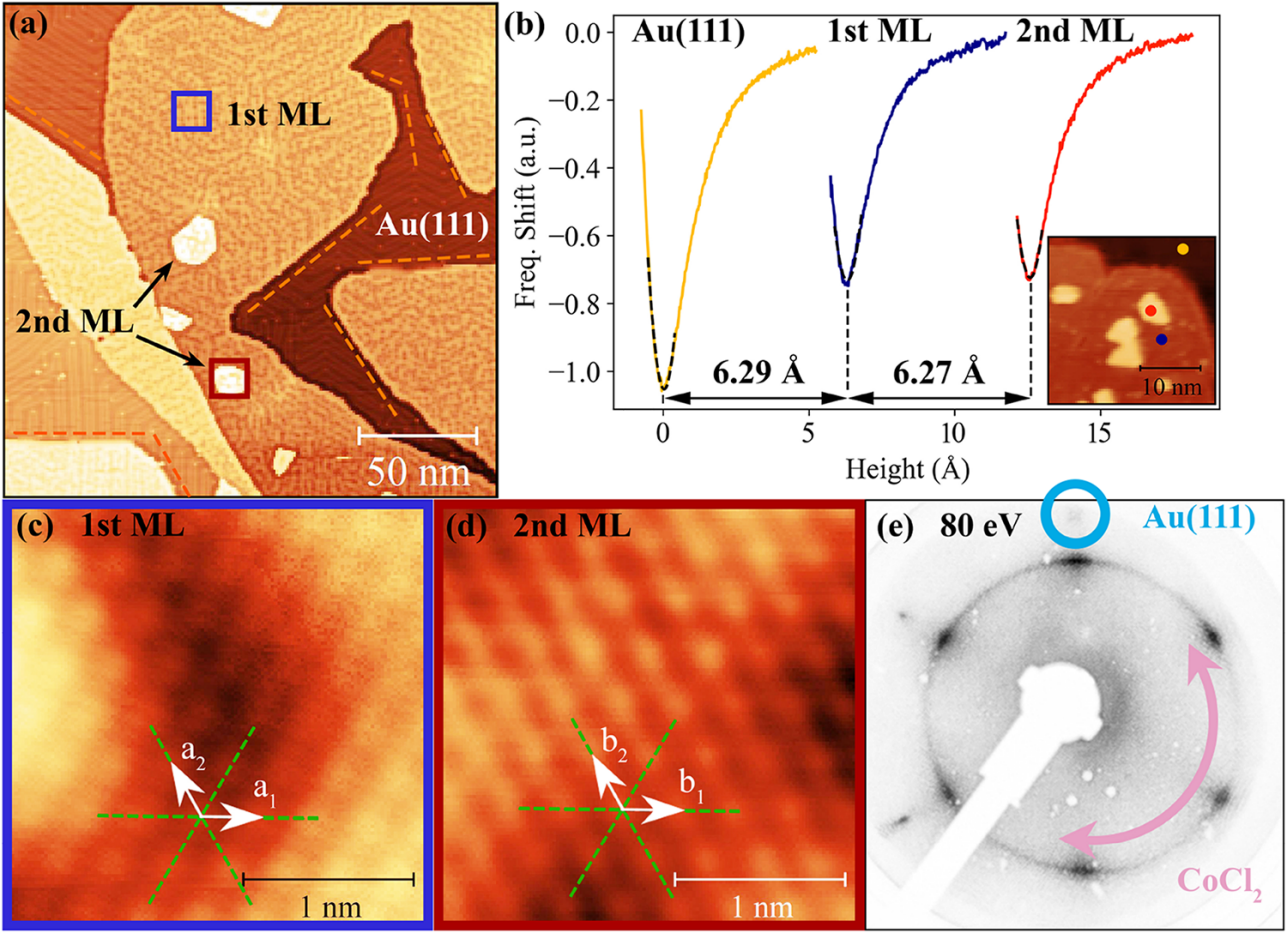
(a) Large-scale STM image showing the growth of first-layer CoCl_2_ islands and the initiation of second layer growth in the sub-ML coverage regime. The hexagonal symmetry of the first layer is suggested by the straight edges marked by orange dashed lines. (b) Height measurements via Z-spectroscopy with a CO functionalized tip. Atomic resolution of (c) the first and (d) the second layer shown on the same absolute color scale. All unit vectors (*a*_1_, *a*_2_, *b*_1_, *b*_2_) align with the Au(111) lattice (green dashed lines). The ring-shaped CoCl_2_ diffraction pattern (e) with increased intensity along the gold direction suggests the presence of multiple rotational domains that preferentially align with the Au(111) lattice. (a) *U* = 1.3 V, *I*_t_ = 30 pA, 210 nm, nominal coverage = 0.8 ML, (c) *U* = 0.2 V, *I*_t_ = 27 pA, 5 nm, *a*_1_ = *a*_2_ = 3.6 ± 0.1 Å, (d) *U* = 0.8 V, *I*_t_ = 20 pA, 5 nm, *b*_1_ = *b*_2_ = 3.6 ± 0.1 Å, (e) LEED: 80 eV.

CoCl_2_ exhibits a regular ABC stacking order in bulk with parallel aligned atomic rows between the layers, so we expect a similar behavior when growing this material on Au(111).^25^ This can be seen in the second-layer islands, which appear consistently aligned with the first-layer island (Figures 1 and S5). In contrast, first-layer islands can have any orientation with respect to Au(111), again revealing the low TMDH/Au(111) interaction.^26,27^ The diffraction pattern of a LEED measurement in Figure 1e is an average of all periodic structures in the topmost few layers of the sample (highly surface sensitive). Since the Au(111) crystal has a fixed orientation and hexagonal symmetry, it creates only one set of hexagonal diffraction spots at a fixed position in reciprocal space. A ring-shaped LEED pattern on the other hand indicates that the atomic rows can have any orientation relative to the substrate. Low-energy electron diffraction (LEED) (Figure 1e) reveals a slight preference aligning with the close-packed Au(111) directions, evident from the enhanced intensity in the ring-shaped pattern. An increase in coverage shows a weakening of the Au(111) spots and no appearance of new diffraction spots (see Θ = 3.2 ML, Figure S11), indicating that the CoCl_2_ structure remains the same.

According to high-resolution STM images, the lateral lattice constant for the first and second layers (Figure 1c,d) is 3.6 ± 0.1 Å (determination in Figure S4), which is in good agreement with the 3.54 Å predicted in literature.^10^ To obtain the interlayer distances, Z-spectroscopy via noncontact AFM with a CO functionalized tip was performed (Figure 1b). By approaching the tip while measuring the frequency shift, the point of equilibrium between attractive and repulsive forces is identified, enabling the determination of the height between layers. A step height of approximately 6.3 Å is obtained for the first and second layers, close to the 5.93 Å predicted for CoCl_2_’s 1T structure in literature.^28^ 1T-CoCl_2_ consists of three atomic planes stacked in a Cl–Co–Cl manner (Figure S1). Since X-ray photoelectron spectroscopy (XPS) measurements revealed a Co to Cl ratio of 1:2 and consistent peak positions across different coverages (see Figure S11), we confirm regular and stoichiometric bulk-like growth.^29^ Theoretical calculations for the closely related FeCl_2_ have shown that the 1T configuration is the most stable and energetically favorable.^30,31^

Figure 2a shows a typical U-shaped differential conductance curve, measured on the first CoCl_2_ layer, which suggests an ≈3.9 eV band gap.^31–33^ Closer inspection of the bias range from −1 to 1 V reveals states appearing within the band gap of CoCl_2_. Figure 2a (inset) shows an STS color map obtained by measuring point spectroscopy on 25 consecutive locations along the dotted line (Figure 2b,c). Four selected point spectra measured at different positions (point numbers: 2, 8, 16, and 24) illustrate the spectral changes of the states in this reduced voltage range. To better visualize the spatial distribution of these in-gap states, d*I/*d*V* maps (*V*_Mod_ = 5 mV) were measured at four bias values of interest marked by the white, dotted, vertical lines in Figure 2a. d*I*/d*V* maps measured at these bias voltages shown in Figure 2d–g directly display the spatial distribution of these states on the surface. In this direct intensity comparison, the map at 0.4 V in Figure 2f shows almost no contrast, as no states are visible across the entire range of the point spectra (see 0.4 V in the color map). Figure 2d,e,g exhibits modulation in the spatial distribution of the states at −0.18, −0.05, and 0.80 V, respectively.

**Figure 2:**
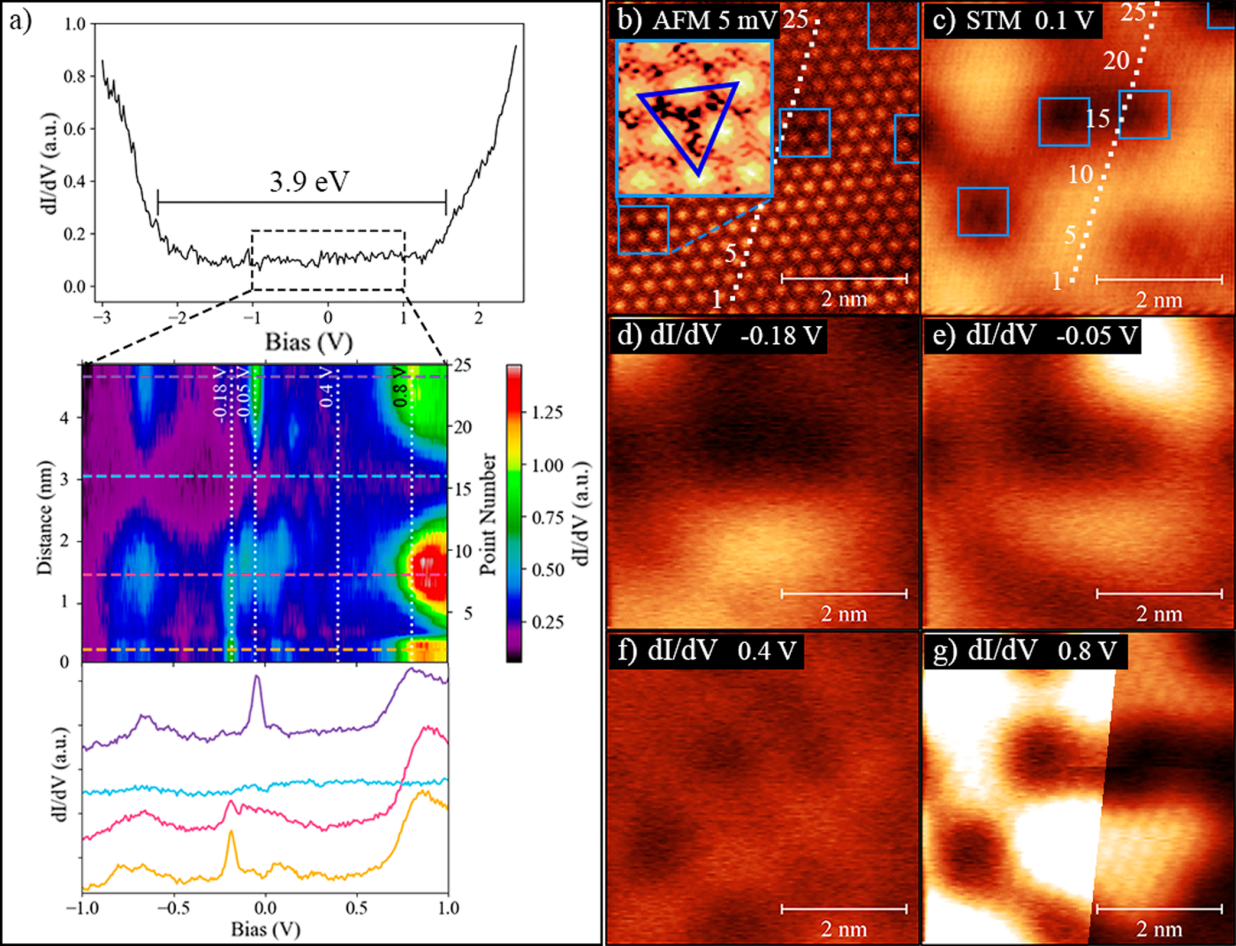
(a) d*I*/d*V* spectrum showing the onset of the valence and conduction band of CoCl_2_. Twenty-five d*I*/d*V* spectra measured within the bias range from −1 to +1 V are shown in the inset. Four representative spectra are shown below the color map, corresponding to the color-coded horizontal lines (point numbers 2, 8, 16, 24). (b, c) AFM and STM images indicating the positions of the 25 d*I*/d*V* measurements. d*I*/d*V* maps at constant current (*I*_t_ = 10 pA); (d) −0.18 V, (e) −0.05 V, (f) 0.4 V, and (g) 0.8 V analyze the spatial distribution of the in-gap states within a ±5 mV range on the same absolute color scale (in (g) left: same color scale; right: adjusted color scale for improved visualization). The blue squares in (b) and (c) indicate areas with structural alterations from the normal atomic resolution with hexagonal symmetry causing a contrast depletion centered between 3 top-layer Cl atoms (blue triangle).

The emergence of these states is clearly position-dependent and does not follow the atomic periodicity of the layer, visible in Figure 2c. Upon closely analyzing the atomic resolution in the AFM image in Figure 2b (inset), a contrast depletion centered between three top-layer Cl atoms is visible (blue triangle in the inset, also marked by blue squares at other sites in the image). This depletion coincides with the darker regions observed in STM (c) indicating areas with a reduced local density of states (LDOS). Note the absence of in-gap states in the proximity of these reduced LDOS sites across the entire bias range. Locally stronger interactions between a bottom layer Cl atom and a Au(111) atom due to their incommensurate lattices or subsurface defects could be respo this feature. The large-scale electronic contrast observed in STM measurements of the second monolayer is notably weaker (see Figure 1c,d: same absolute color scale). Nevertheless, the in-gap states can be clearly observed in the STS spectra measured in the bilayer (see Figure S7).

To analyze surface state dispersions, a series of d*I*/d*V* maps was measured over a bias range from −0.45 to +0.50 V on both clean Au(111) and CoCl_2_-covered regions (Figure 3). Applying a Fourier transform to the d*I*/d*V* maps measured at different bias voltages allows for the visualization and characterization of the parabolic dispersion of the surface state. The CoCl_2_ film modifies the surface state of the Au(111) substrate, typically located at −0.5 V,^34^ by shifting it to higher energies. This shift occurs because the CoCl_2_ layer acts as a barrier, compressing the Au(111) surface state into a more confined space and thereby raising its energy. This is shown in Figure 3a, where each energy shows the radial distribution of these states in *k*-space. Figure 3b,c shows the d*I*/d*V* map and its corresponding fast Fourier transformation (FFT) at 0.3 V. Note how the states marked by the white and violet dashed lines in Figure 3a are also visible in FFT (c-arrows). The experimental analysis follows previous work^34,35^ and is detailed in Figure S8. The dispersion relation for these surface states can be described by the equation:

1 $$E\left(k\right)=E_{0}+\frac{\hbar^{2}k^{2}}{2m^{*}}$$

where *E*(*k*) is the energy of the electrons as a function of the wave vector *k*, *E*_0_ is the energy at the minimum of the parabola, ℏ is the reduced Planck’s constant, and m* is the effective mass of the electrons.

**Figure 3:**
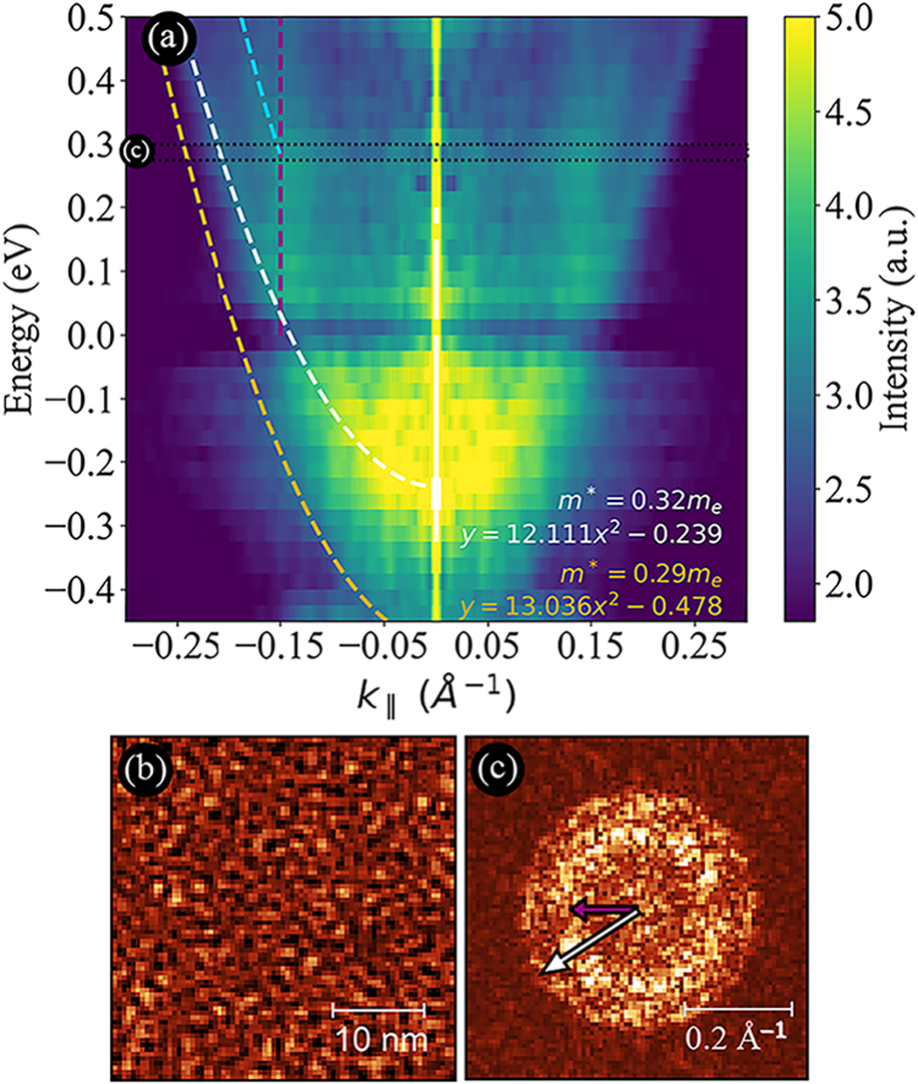
(a) Dispersion relation of CoCl_2_ on Au(111) extracted from the d*I*/d*V* maps measured in the bias range from −0.45 to +0.5 V. The white parabola represents the dispersion of the interface state on the CoCl_2_-covered region, which is shifted to higher energies compared with the pristine Au(111) surface. The orange parabola, overlaid schematically, indicates the dispersion of the clean Au(111) surface state (Figure S10) and the light blue dashed line shows a second additional dispersive feature we were not able to identify. A nondispersive feature, present only in the positive bias voltage range, that Schouteden et al.^34^ attributed to the Au(111) bulk states is marked by a violet dashed line. This nondispersive feature is found at 0.15 ± 0.01 Å^–1^, which corresponds to a 21 Å distance in real space (Figure S9). The horizontal black dotted line (labeled as (c) on the left side) corresponds to the energy range in the dispersion plot related to the Fourier transform shown in (c). (b) d*I*/d*V* map of CoCl_2_ on Au(111) acquired at a sample bias of 0.3 V with a modulation amplitude of 5 mV. (c) Fourier transform of the real-space d*I*/d*V* map, highlighting two concentric rings as indicated by the color-coded arrows.

By fitting the parabolic dispersion using eq 1, the effective mass of electrons on both the pristine Au(111) surface and the CoCl_2_-covered regions can be determined (see Figure 3). The effective mass for the clean Au(111) surface was determined at *m*_Au(111)_^*^ = 0.29*m*_e_ (see Figure S10), while for the CoCl_2_-covered regions, it amounted to *m*_CoCl_2_/Au(111)_^*^ = 0.32*m*_e_. This slight increase in effective mass in the CoCl_2_-covered regions suggests that the CoCl_2_ film introduces minimal scattering or interaction effects, maintaining the nearly free-electron-like nature of the surface state.

Another feature of interest visible only in the positive bias voltage range in Figure 3 is denoted by the violet dashed lines at ±0.15 Å^–1^ and appears to be nondispersive. This feature has been previously interpreted as resulting from gold bulk state electrons contributing to the local density of states.^34^ Such nondispersive behavior, especially in the energy range above the Fermi level, contrasts with expectations from the projected band structure of bulk gold. The anomaly is likely due to differing inelastic relaxation rates for electron states above and below the Fermi level, which may cause a constant wave vector for bulk state electrons above the Fermi level.^34^ The feature is very weak, likely due to tunneling through the CoCl_2_ film, although alternative origins cannot be ruled out.

Once the structural and electronic properties of this single CoCl_2_ layer are experimentally determined, we proceed to study its magnetic character. CoCl_2_’s magnetic properties were measured via XAS/XMCD using circularly polarized synchrotron X-ray radiation. In contrast to conventional sample preparation, different coverages were all achieved on a single sample producing a gradient by systematically positioning a shutter across the evaporation beam, effectively covering distinct portions of the surface (see Figure S2 and Methods Section). Figure 4 shows measurements of CoCl_2_ at different coverages (0.4, 1.1, and 1.4 ML) under both normal (NI) and grazing (GI) X-ray beam incidence. Upon examining the XAS spectra shown in Figure 4a and, in particular, the magnetization loop illustrated in Figure 4c, it becomes immediately apparent that the easy axis in CoCl_2_ lies in the plane, signaling in-plane magnetization. Under grazing incidence, the magnetization loop achieves full saturation at magnetic field strengths below 1 T. Conversely, in normal incidence, the magnetization loop is practically a straight line with a shallow slope, failing to saturate even at 6 T. Since the NI measurements show no evidence of saturation at 6 T, the anisotropy field can be estimated extrapolating the NI and GI magnetization curves to their point of intersection. This leads to a saturation field of 31.4 T and corresponds to an anisotropy energy of 1.6 meV per Co atom (*H*·μ_*at*_/2 = *E*_a_,^36^ where we use a spin atomic moment μ_at_ of 1.81 μ_B_). It compares well to the value obtained for the monolayer CoCl_2_ by the DFT calculations (0.7 meV^31^). On the other hand, much weaker anisotropy fields of 3 and 0.5 T were found for the Ising-type ferromagnets CrI_3_ and CrBr_3_, respectively.^10,37,38^ This high saturation field indicates a robust in-plane anisotropy, emphasizing the distinctive magnetic behavior of CoCl_2_ compared with other TMDH.^16,19^ Another key feature can be inferred from the XAS and XMCD peak structure in Figure 4a,b: The multiplet splitting at the Co L_3_- and L_2_-edge remains consistent with increasing coverage. This feature suggests a uniform and single-phase growth that maintains its characteristics between the first and second monolayer and is not affected by the theoretically predicted antiferromagnetic coupling between layers.^31^ This also points to minimal interactions between the Au(111) substrate and CoCl_2_, further reinforcing the conclusions about the weak interlayer forces and strong intralayer stability discussed previously.

**Figure 4:**
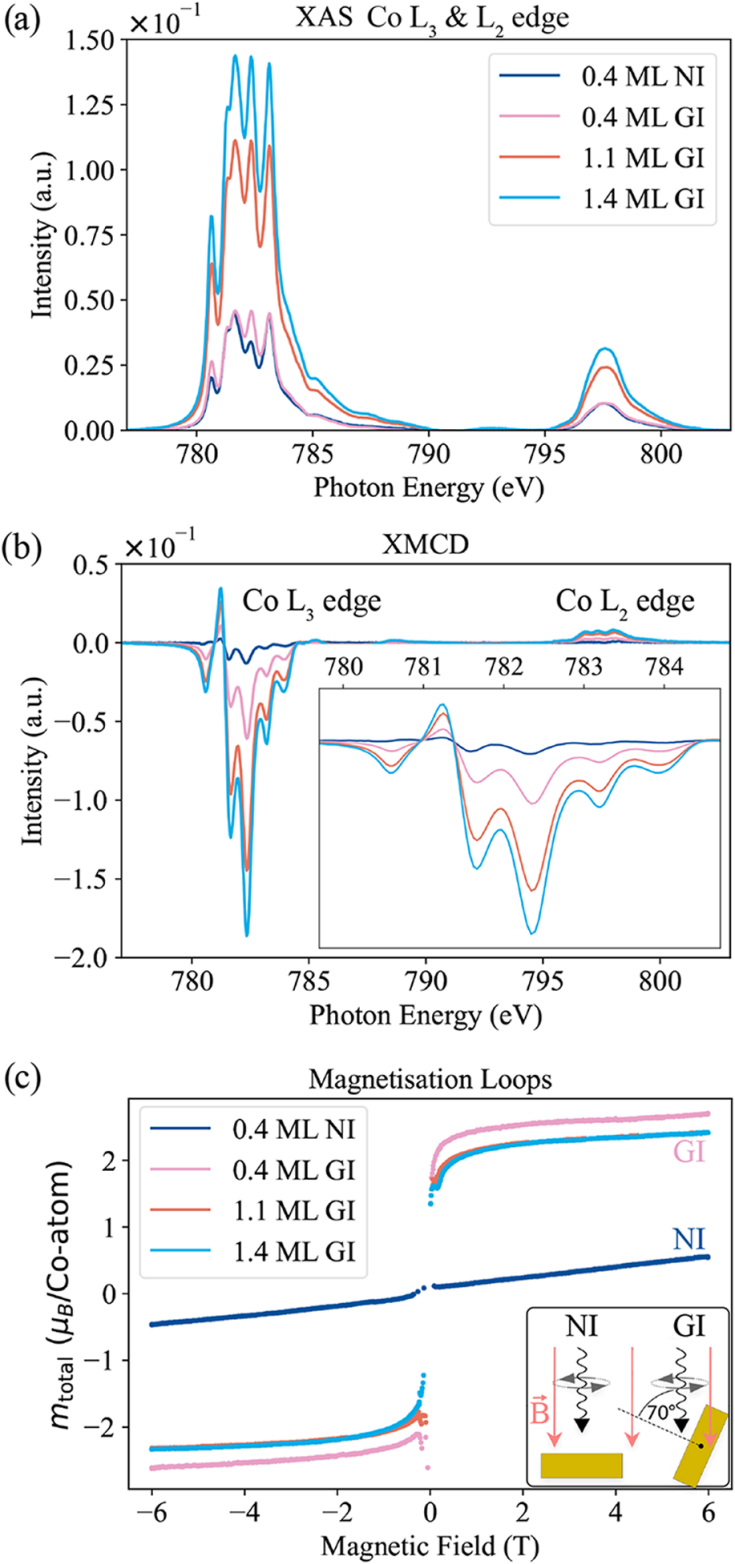
Magnetic characterization of CoCl_2_ on Au(111). (a) XAS and (b) XMCD spectra measured at 2 K with an applied field of 6 T measured at the Co L_3_- and L_2_-edge in NI (normal incidence, 0°) and GI (grazing incidence, 70°) are displayed for different coverages of CoCl_2_ on Au(111). The XMCD spectra are scaled to the peak height of the average XAS spectra. (c) Magnetization loops measured at the L_3_-edge, for the 0.4 ML sample in NI and GI and the 1.1 and 1.4 ML sample in GI. All artificial spikes around 0 T have been removed because fluctuations in the magnetic field or alignment imperfections near zero can cause inconsistencies in total-electron-yield (TEY) measurements.

After investigating the behavior of CoCl_2_ for different thicknesses, the temperature-dependent magnetization loops were explored. This involved incrementally adjusting the system’s set point temperature, while the magnetic field strength was varied from −6 to +6 T and back. Four distinct temperatures were studied: 2, 8, 15, and 25 K. In Figure 5a, a noticeable and gradual transition is observed in the magnetization loop of 1.4 ML CoCl_2_ from an almost step-like profile at 2 K to a flatter, s-shaped curve at 25 K (T-dependent loops at 0.4 and 1.1 ML are plotted in Figure S13). We used Arrott plot analysis to determine the critical temperature as in refs 17,39 and found a transition temperature of 24 K. Below the transition temperature, CoCl_2_ shows ferromagnetic properties, with the magnetic moments aligning to produce a step-like profile in the magnetization loop. As the temperature rises above 24 K, CoCl_2_ becomes paramagnetic, indicated by a more gradual, s-shaped magnetization curve. This shift reflects the loss of magnetic order due to thermal excitation, causing the magnetic moments to orient randomly without any long-range alignment.

**Figure 5:**
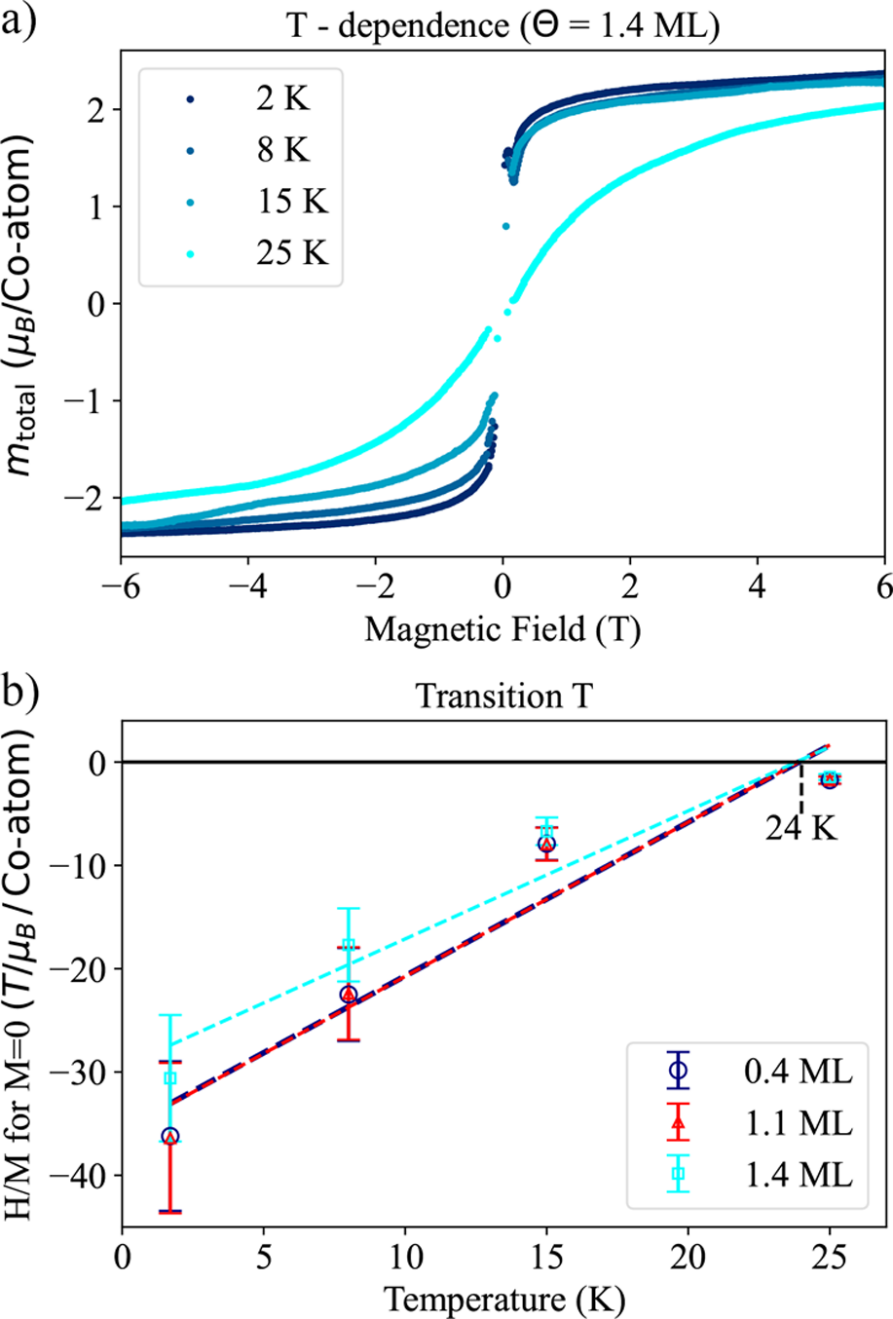
(a) Temperature-dependent magnetization loops of the 1.4 ML CoCl_2_ sample. Notice how the curves shift from resembling an almost step function to an S-shaped pattern as the temperature increases. (b) Linear regression of the points obtained via the temperature-dependent magnetization loops and Arrott plots (see Figure S13) at each system temperature (1.7, 8, 15, 25 K). The intersect of the linear regression with the *x*-axis at 24 K denotes the transition temperature from a ferromagnetic to a paramagnetic order. The 0.4 and 1.1 ML linear regressions coincidentally overlap.

We calculate spin and orbital magnetic moments via sum rules analysis (Figure S13): Halogens are generally considered to create a weak crystal field, thus leading only to a weak energy difference between *e*_g_ and *t*_2g_,^40^ leading predominantly to a high-spin configuration. Co^2+^ being a 3d^7^ transition metal would, according to Hund’s rules, have a magnetic moment of 3μ_B_ per Co atom. At the lowest temperature (2 K), the calculated magnetic moments of CoCl_2_ for all measured coverages are lower than those predicted by Hund’s rules (see Table 1). This was observed for the whole family of TMDH,^16,19^ besides NiBr_2_ on Au(111).^17^

**Table 1:** Magnetic Moments Measured by XMCD for Different Coverages (Θ) at *T* = 2 K and *B* = 6 T^a^

		μ (μ_B_/Co atom)			
		NI	GI		
Θ (ML)	*T* (K)	*m*_tot_	*m*_s,eff_	*m*_l_	*m*_tot_
0.4	2	0.52	1.81	0.91	2.72
	8		1.76	0.89	2.65
	15		1.79	0.87	2.66
	25		1.34	0.76	2.10
1.1	2	0.52	1.60	0.81	2.41
	8		1.63	0.78	2.41
	15		1.62	0.76	2.38
	25		1.41	0.67	2.08
1.4	2	0.54	1.60	0.83	2.43
	8		1.53	0.79	2.32
	15		1.52	0.77	2.29
	25		1.35	0.70	2.05

a The magnetic moments (μ) are calculated for the in-plane and out-of-plane measurements. The total magnetic moment *m*_tot_ per Co atom is the sum of the effective spin moment *m*_s,eff_ and the orbital moment *m*_l_. The measured moments are shown for NI and GI with an error of ±10%. More details about the procedure of sum rules analysis are available in Figure S13.

The reduced magnetic moments observed in CoCl_2_ and other TMDHs could potentially be attributed to a combination of geometric and electronic factors. In CoCl_2_, cobalt atoms are located in the center of an octahedral arrangement of chloride ions. Owing to its strong ionic nature, the cobalt-chloride distance is mostly determined by the size of the Cl^–^ anion. This could reduce the orbital overlap between the cobalt d-orbitals and chlorine p-orbitals, leading to a weaker crystal field. The presence of lattice distortions due to interactions with Au(111) might also affect the electronic configuration, potentially allowing for a mixture of high-spin and low-spin states. Deviations from a perfect octahedral symmetry can further split the *t*_2g_ and *e*_g_ orbitals into additional sublevels. These distortions could lead to partial quenching of the orbital magnetic moment, which, through spin–orbit coupling, can also reduce the effective spin magnetic moment. Both the quenching and the modified orbital environment could influence the magnetic anisotropy, as seen with the strong in-plane magnetization in CoCl_2_. This suggests that the reduced magnetic moments in CoCl_2_ and similar TMDHs could result from reduced orbital overlap, modified crystal field effects, altered electronic configurations, and inhomogeneities in the lattice.

## Conclusions

CoCl_2_ on Au(111) exhibits highly ordered growth with weak substrate interactions carpeting over Au(111) step edges, demonstrating a high intralayer stability. Similar to the bulk material, it shows a semiconducting behavior with a band gap of approximately 3.9 eV. Surprisingly, in-gap states near Fermi energy arising from interface interactions with gold were measured via STS, opening possible applications in interface engineering, tunable charge transport, or enhanced catalytic activity.

XAS and XMCD measurements also reveal a strong in-plane magnetic anisotropy and a high transition temperature of around 24 K from ferromagnetic to paramagnetic ordering. The magnetic moments are lower than predicted by Hund’s rules, which may be attributed to geometric and electronic factors that lead to decreased orbital overlap, altered crystal field effects, changes in electronic configurations, and partial quenching of the magnetic moment. These findings offer new ways for tailoring magnetic properties through interface engineering or substrate choice. The unique combination of semiconducting behavior, interfacial gap states, and magnetic ordering positions CoCl_2_ as a promising material for next-generation nanoscale devices. Applications in spintronics, magnetic sensors, or energy-efficient memory technologies are particularly compelling, given its potential for tunable electronic and magnetic properties.

## Methods

Cobalt dichloride (CoCl_2_) films (submonolayer to multilayer) were grown on Au(111) using a Knudsen cell evaporator with quartz crucibles (Dodecon OMBE Source^41^). The evaporation was performed under ultra-high vacuum (UHV) conditions (evaporation pressure of 10^–8^–10^–9^ mbar) at a temperature of roughly 400 °C. While FeBr_2_ has been shown to grow with the substrate at room temperature,^16^ slightly elevated substrate temperatures of roughly 100 °C were needed for ordered CoCl_2_ growth, analogue to FeCl_2_ and NiCl_2_.^19^ The amount of evaporated material was estimated via a quartz microbalance, while low-temperature scanning tunneling microscopy (LT-STM), X-ray photoelectron spectroscopy (XPS), and low-energy electron diffraction (LEED) were used as a cross-reference.

The purity of the anhydrous beads of CoCl_2_ from Sigma-Aldrich used in the experiment was labeled at 99.9%^42^ and the substrate cleaning process involved standard Ar^+^ sputtering and annealing cycles at 720 K.

The CoCl_2_ films (from 0.4 ML, 1.1, and 1.4 ML) used for XAS experiments were prepared consecutively on the same sample by incrementally adjusting a shutter across the Au(111) crystal at predefined intervals. This yields a sample with a gradient of coverage grown sequentially. A schematic representation is shown in Figure S2.

The magnetic characterization was performed at the DEIMOS beamline at the SOLEIL synchrotron with a degree of circular polarization of 98%.^43^ The lowest achievable measurement temperature was 2 K^44^ and higher system temperatures were attained through counter heating and thermostatization. The sample’s circular dichroism was measured in normal incidence (NI) as well as in grazing incidence (GI ∼ 70°), as is shown in Figure 4, yielding information about the material’s anisotropy and magnitude of the magnetic moment. Table 1 contains the magnetic moment values obtained from the sum rule analysis for the different sample coverages and temperatures are displayed.

Figure 4a shows the white line absorption spectra at the Co L_3_- and L_2_-edges of Co, averaged over both left and right circular polarizations. Figure 4b displays the corresponding XMCD spectra, and Figure 4c presents the magnetization loops measured at a beam energy of approximately 782.5 eV, where the XMCD signal reaches its maximum. The XAS spectra have been normalized to the pre-edge and a background subtraction has been performed.^45^ The magnetization curves measured for different coverages at ≈2 K in the two different geometries were measured in total electron yield (TEY) mode and are scaled to the calculated expectation values of the total magnetic moment at 2 K and 6 T. As a result, the intensity values correspond to the projection of the total magnetic moment per Co atom in the direction of the X-ray beam under a 6 T magnetic field.

The STM experiments were performed with a Scienta-Omicron LT-STM at 4.3 K and 10^–10^ mbar base pressure.

The AFM experiments were performed with a SPECS Low-temperature Scanning Probe Microscope with Joule-Thompson stage at 1.4 K and a CO-functionalized tip with Kolibri sensor.^46^ The base pressure was 10^–10^ mbar and Nanonis software was used.

The XPS measurements were carried out with a Phoibos 100 photoelectron spectrometer, using a nonmonochromatic Al Kα X-ray source. The energy resolution is 0.1 eV.

UHV conditions were preserved during all of the sample transfers (base pressure during the experiment was 10^–10^ mbar).

## Supplementary Material



## Notes

Schematic Structure, Preparation of Wedged Sample, STM Length and Rotation Calibration, Large-Scale STM of 1–1.5 ML CoCl_2_ Coverage, STS in the Second ML, Surface State Dispersion Calculation, FFT and Au(111) Dispersion, XPS Fitting Procedure and Parameters, XPS Co 2p, Cl 2p, Au 4s, and Survey Plot, LEED Series up to 3.2 ML, XAS Coverage Calibration, XMCD Sum Rule Analysis, Temperature-Dependent Magnetization Curves and Arrott Plots (PDF)
