# Supplementary material for: Strong In-plane Magnetic Anisotropy in Semiconducting Monolayer CoCl2
Source: ACS Nano. 2025 May 30;19(22):20693–701. doi: 10.1021/acsnano.5c02175 (PMC12164524; doi:10.1021/acsnano.5c02175)
Supplement: Supplementary file 1 [file nn5c02175_si_001.pdf]

Strong In-plane Magnetic Anisotropy in Semiconducting Monolayer $CoCl_2$

Samuel Kerschbaumer [*,†] Sebastien Elie Hadjadj [†] Andrea Aguirre-Baños [†] Danilo Longo [‡] Andrés Pinar Solé [¶] Oleksandr Stetsovych [¶] Adriana Elizabeth Candia [†,§] Paula Angulo-Portugal [†] David Caldevilla [†] Fadi Choueikani [‖] Martina Corso [†] David Serrate [⊥,#] Jorge Lobo-Checa [⊥,#] Pavel Jelínek [¶] Maxim Ilyn [†] and Celia Rogero [*,†]

†*Centro de Física de Materiales (CSIC/UPV-EHU), 20018 Donostia-San Sebastián, Spain*

‡*CIC nanoGUNE-BRTA, 20018 Donostia-San Sebastian, Spain*

¶*FZU - Institute of Physics of the Czech Academy of Sciences, Cukrovarnická 10, Prague 6, CZ 16200, Czech Republic*

§*Laboratorio de Microscopias Avanzadas (LMA), Universidad de Zaragoza, Zaragoza E-50018 Spain*

‖*Synchrotron SOLEIL, 91190 Saint-Aubin, France*

⊥*Instituto de Nanociencia y Materiales de Aragón (INMA), CSIC-Universidad de Zaragoza, 50009 Zaragoza, Spain*

#*Departamento de Física de la Materia Condensada, Universidad de Zaragoza, E-50009 Zaragoza, Spain*

E-mail: kerschbaumersamuel@gmail.com; celia.rogero@ehu.eus

Phone: +34 943 01 5804

Supplementary Information

Schematic Crystal structures

Figure 1 presents the top and side views of the octahedral structure of $CoCl_2$. The system exhibits lattice constants of a = b = 354 pm and a c-lattice constant of 593 pm.[1] Co atoms are represented in blue, while Cl atoms are shown in yellow. In the lower right, the 1T-structure of $CoCl_2$ is depicted, showcasing the characteristic 180° rotated Cl-planes.

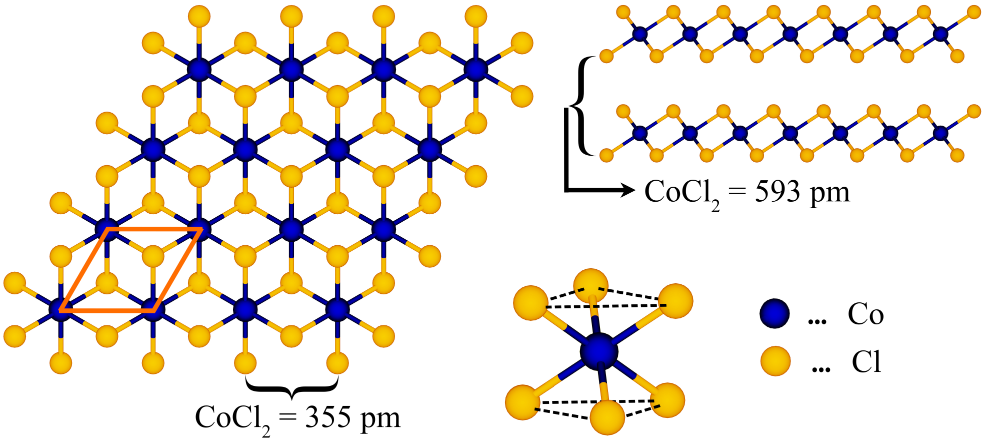

Figure 1: Schematic representation of $CoCl_2$'s trilayer structure. The layers are composed of triangular lattices of cations arranged in edge-sharing octahedral coordination with a 1T (or D_{3d}) - MX_2 structure, creating a configuration where the transition metals are sandwiched between two halide layers.

Wedged Sample Preparation

The X-ray Absorption Spectroscopy (XAS) sample of $CoCl_2$ (0.4 ML, 1.1 ML and 1.4 ML) was prepared consecutively on the same sample by incrementally adjusting a shutter across the Au(111) crystal at predefined intervals. This yields to a sample with different coverages along the crystal. From an experimental point of view, this sample preparation technique eliminates numerous potential sources of error, such as variations in sample positioning and evaporation conditions, as only the shutter requires movement or adjustment. Furthermore, this approach results in a substantial reduction in measurement time, as there is no need to

cool the sample to 2 K for each new coverage iteration. Figure 2 illustrates the experimental setup, highlighting the streamlined process. The distinct regions of different coverage were clearly discernible, thus allowing the positioning of the X-ray beam needed for measurement on each coverage individually.

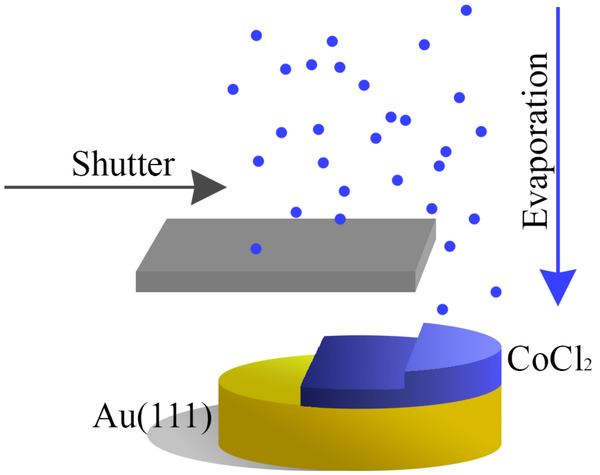

Figure 2: Throughout the evaporation process, a shutter is incrementally shifted across the Au(111) sample at defined intervals, resulting in distinct regions of varying coverages on the same substrate. The physical constraints of the crystal and the X-ray beam diameter, limit the achievable coverages to three on a single 0.5×0.5 cm Au(111) sample.

STM - Determination of the lattice constant and rotation

To determine the lattice constant and rotation of $CoCl_2$ with respect to Au(111), a calibration was performed by means of a Au(111) STM image with atomic resolution. The lattice of Au(111) was measured at 266 pm, which is 8.27 % smaller compared to the 288 pm found in literature.[1] Multiplying all measured lengths by 1.0827 thus yields the calibrated value.

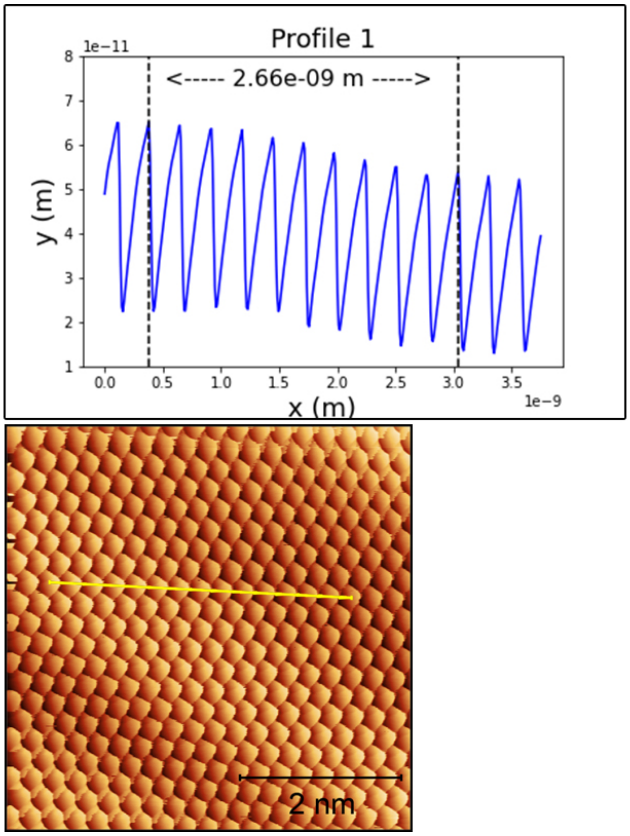

Figure 3: The lattice constant of Au(111) averaged across 10 primitive unit cells was measured at 266 pm. This leads to a calibration factor of 1.0827 when comparing to literature values of 288 pm.[1] $U = -500$ mV, $I_t = 1$ nA.

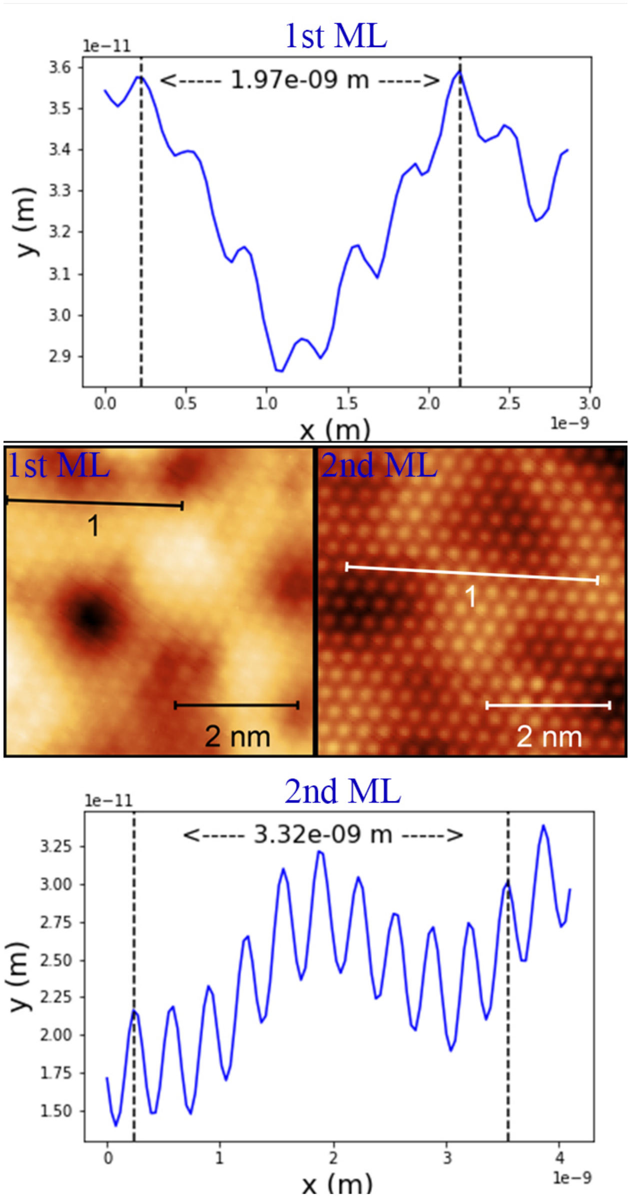

Figure 4: The lattice constant of $CoCl_2$'s 1st ML averaged across 6 primitive unit cells was measured at 328 pm. The lattice constant of $CoCl_2$'s 2nd ML averaged across 10 primitive unit cells was measured at 332 pm. Considering the calibration factor of 1.0827 the measured lattice constant of $CoCl_2$ accounts to 356 pm very close to the literature value of 354 pm. 1st ML: $U = 200$ mV, $I_t = 270$ pA; 2nd ML: $U = 800$ mV, $I_t = 200$ pA.

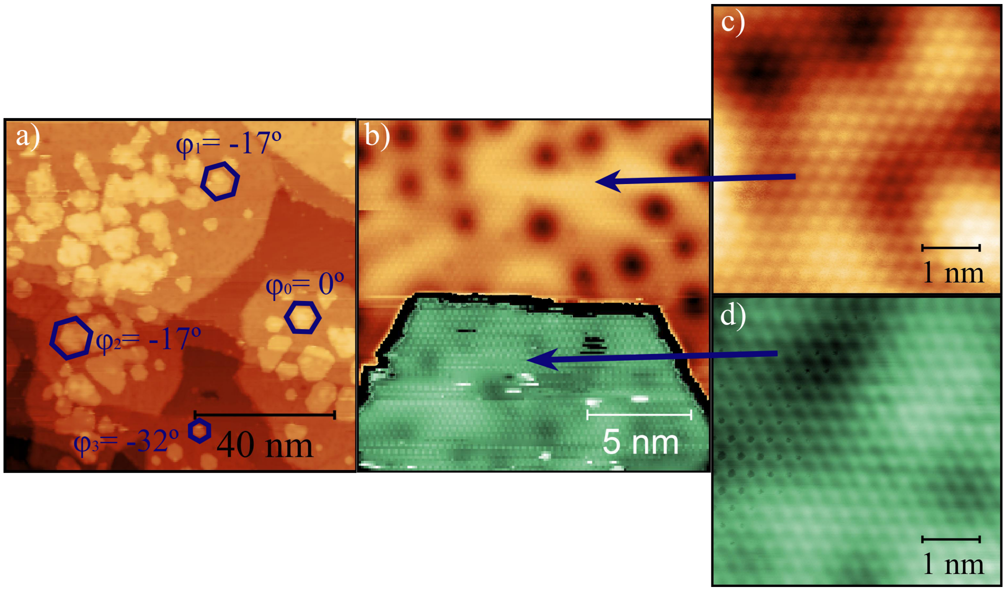

Figure 5: STM measurement showing large scale growth and hexagonal 2nd ML islands growing on the 1st ML. On the same island the hexagonal 2nd layer islands are aligned. $\varphi_1 = \varphi_2$ (most likely the same island overgrowing Au(111) step edges. Note the general hexagonal shape of the 2nd layer island as well as the aligned atomic rows in both layers. (a) $U = 1.45$ V, $I_t = 10$ pA; (b-d) $U = 1$ V, $I_t = 300$ pA.

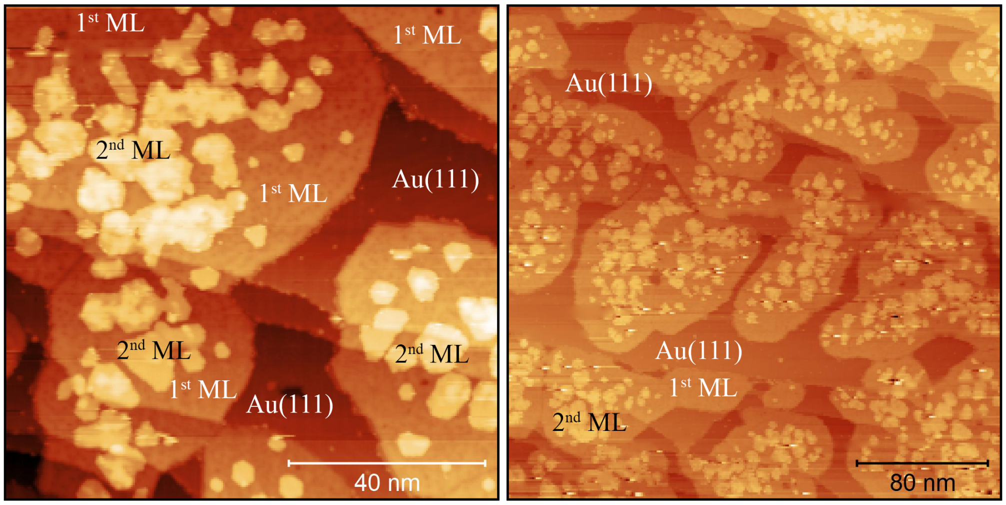

Figure 6: Large scale STM images showing a preparation of approximately 1-1.5 ML. This preparation shows that there is already a considerable amount of second layer growth before the Au(111) surface is fully covered.

STS in the First and Second Monolayer

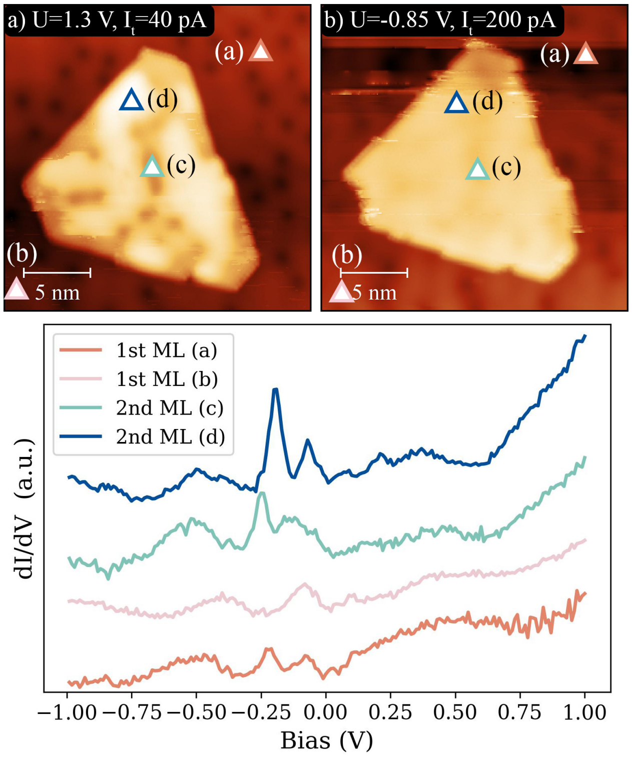

Figure 7: Short range STS on the monolayer and bilayer of $CoCl_2$ with corresponding location on the island measured in STM. The four curves have an offset to improve visibility and are not scaled. STS: $V_{Mod} = 10$ mV

Surface State Dispersion Calculation

The dispersion on both clean Au(111) and $CoCl_2$-covered regions was determined through a series of dI/dV map measurements, taken in 25 mV steps within a range of -450 mV to +500 mV, with a modulation bias of 5 mV. After selecting the relevant area, each dI/dV map was Fourier transformed, and the resulting image was integrated over ϕ, centered at the origin of the FFT, to reduce noise. This process removes any anisotropy information, treating the dispersion as equivalent in both the x and y directions. However, since the nearly free 2D electron gas of the Au(111) surface state exhibits no significant anisotropy, this approach remains valid for dispersion calculations. By plotting the bias voltage (or energy) as a function of the k vector, the surface state dispersion can be observed for both

clean Au(111) and $CoCl_2$-covered regions.

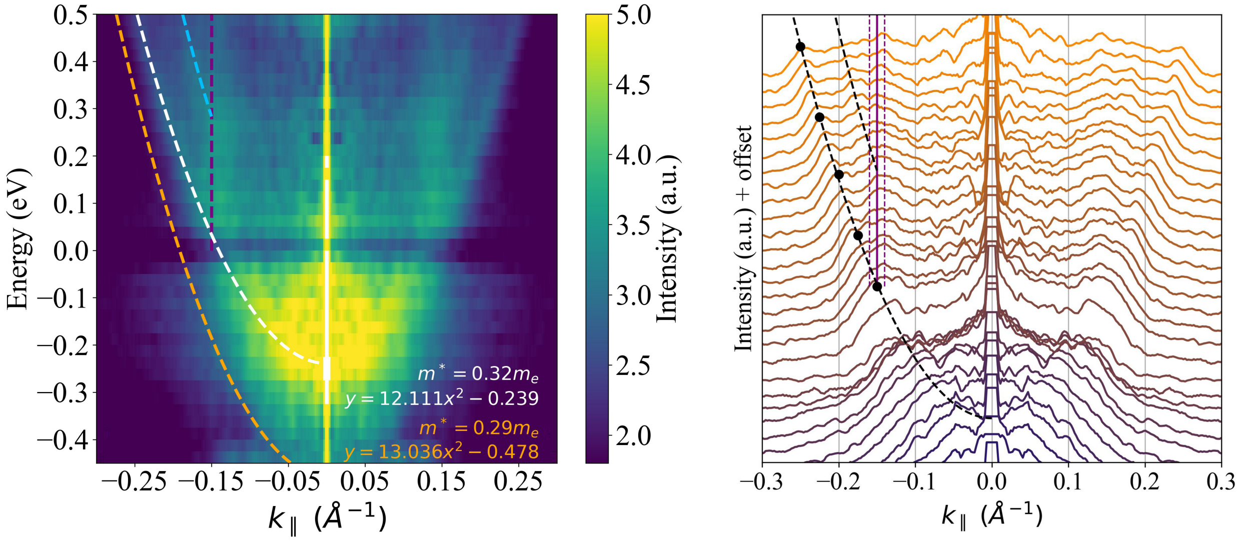

Figure 8: 2D color map and waterfall plot showing the dispersion of the Au(111) surface state on clean Au(111). The orange parabola corresponds to the Au(111) surface state at −0.5 eV, the white parabola indicates the same Au(111) surface state shifted in energy, the light blue dashed line shows a second additional dispersive feature we were not able to identify. The purple line shows a non dispersive feature that Schouteden et. al.[2] attributed to the Au(111) bulk states. The non dispersive feature in the waterfall plot is displayed at 0.15 ± 0.01 $Å^{-1}$ to indicate the peak position.

For $k_\parallel = 0.15$ $Å^{-1}$ a real space distance of 21 Å is obtained. Figure 9 shows the reverse FFT of the inner and outer rings visible in the dI/dV measurement at 0.4 V. Note the similarity to Schouteden et. al.[2]

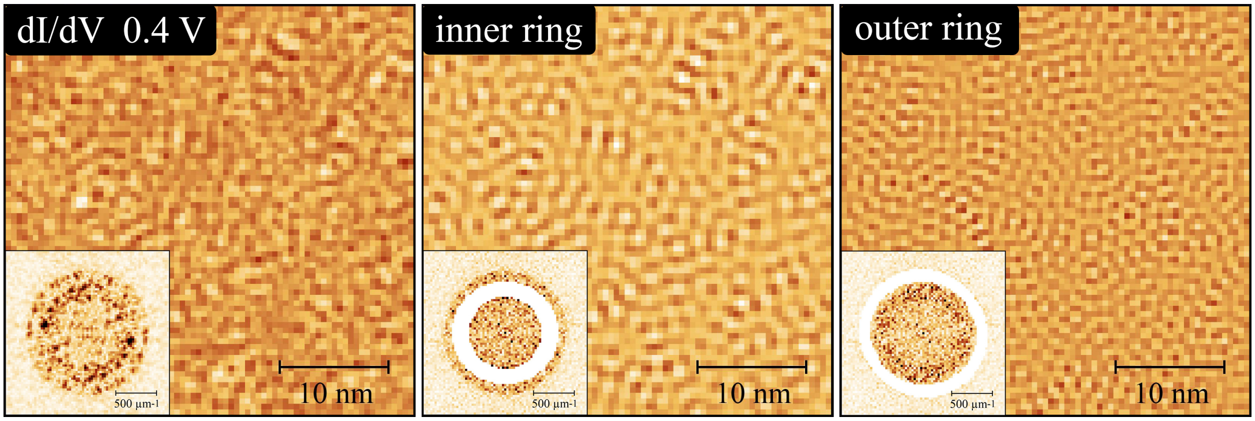

Figure 9: dI/dV map at 0.4 V and inverse FFT of the white-marked region shown in the insets. $V_{Mod} = 10$ mV

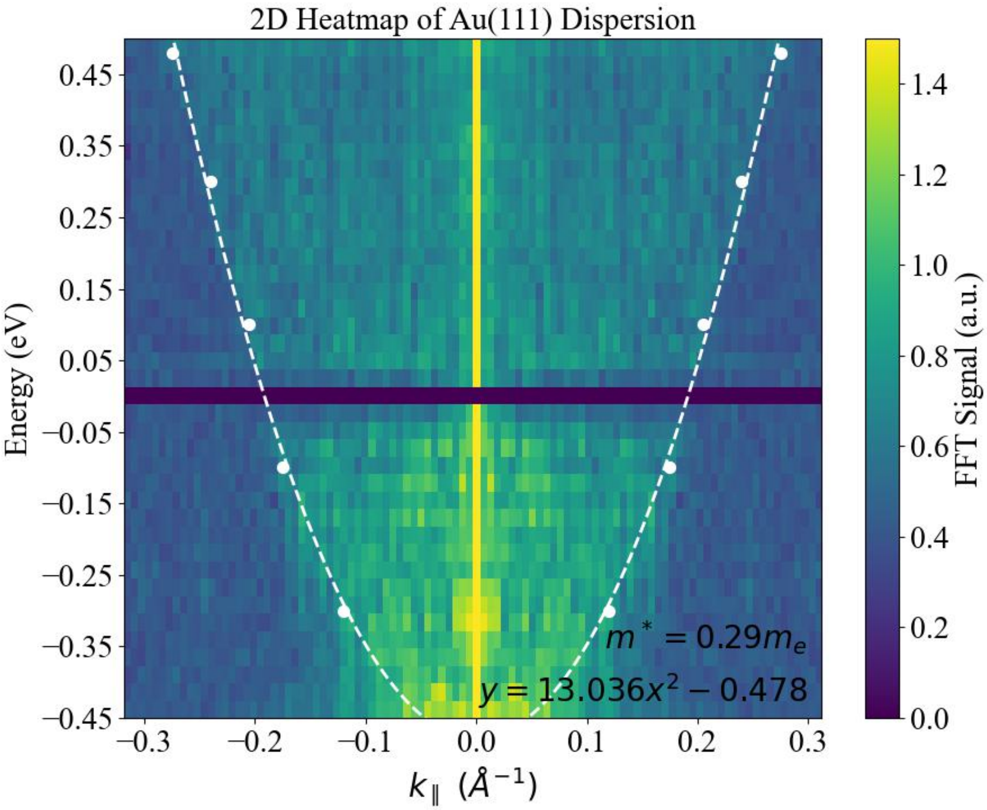

Figure 10: The dispersion of a clean Au(111) region was analyzed, and the parabolic fit yielded a minimum energy of -478 mV, which is close to the known energy of the Au(111) surface state.[2]

XPS fitting procedure and fitting parameter of $CoCl_2$ on Au(111)

In the following the fitting procedure and the resulting fitting parameter for the different coverages of $CoCl_2$ on Au(111) is shown. All Co, Cl, and Au core-level spectra are Shirley background corrected and are fitted by using the lmfit routine.[3] For all spectra Voigt functions have been used to perform the fitting. In the case of the Co fit it was not possible to achieve a fitting error due to the many peaks inside the spectral range. For all fits, we verified that the core-level peaks exhibited the expected area ratios and that the doublet components had equal full width at half maximum (FWHM) values. Table 1 shows the fit parameters for all different coverages of $CoCl_2$ on Au(111). All samples were grown in the

same chamber under the same conditions. The XPS measurements were performed by using a pass energy of 30 eV with the lens setting of Large area (LA). at 1.5 kV and analyser work function of 4.309 eV. The Al-anode with an excitation energy of 1486.61 eV was used and the analyser has an energy resolution of 0.1 eV.

The high intensity of the satellite peaks arises from the greater electronegativity of the ligand (analogue to[4] for $FeBr_2$) compared to, for example, oxygen, and the satellite peaks in the Co 2p region related to the shake-up process occur due to the electron transfer from a 3d orbital to the empty 4s orbital.[5,6] Surface contamination can be excluded, as both survey spectra (see figure 12) and more detailed scans in the oxygen and carbon regions show no increased signal. For the data evaluation, a Shirley background was subtracted, and the data fitting was performed using Voigt profiles from the Python lmfit routine.[3] In total, nine Voigt profiles were needed to fit the Co 2p spectra of $CoCl_2$. The four main peaks include the two Co 2p peaks and the two Co satellite peaks. Since the measurements were performed using the Al-anode of a non-monochromatic gun, four additional peaks related to the anode are visible. The anode-related peaks are shifted by 9.8 eV[7] to lower binding energy with an intensity ratio of 6.4% compared to the real material peaks. The ninth peak is not related to the material but to the substrate (Au 4s). For additional details regarding the fitting parameters, refer to Table 1.

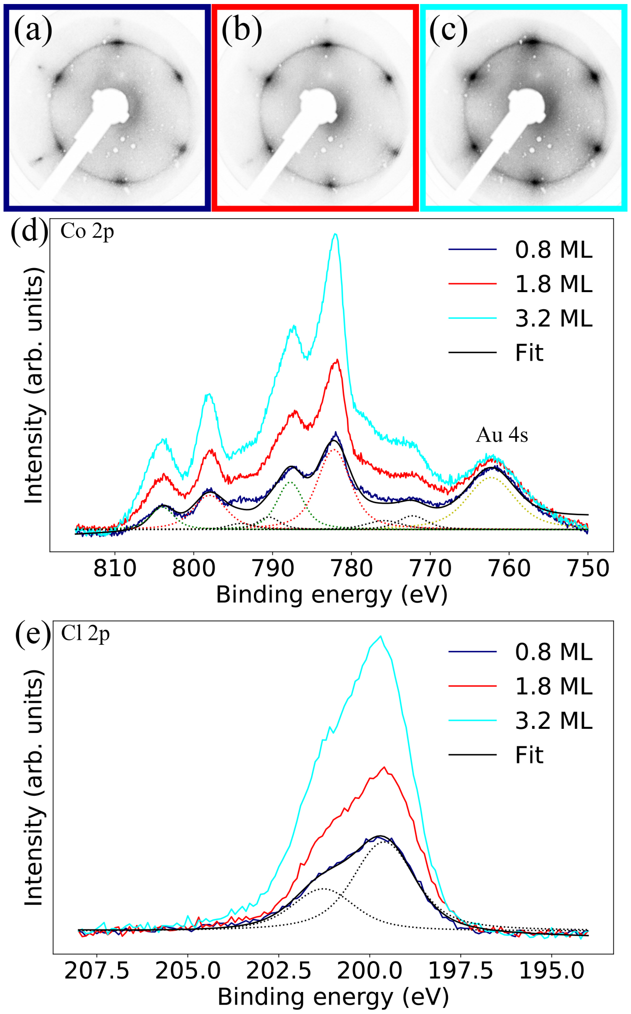

Figure 11: (a) 0.8 ML, (b) 1.8 ML and (c) 3.2 ML show LEED measurements at 80 eV of the respective XPS measurements in (d) and (e). An increase in coverage shows a strengthening of the ring-shaped pattern in LEED attributed to $CoCl_2$, while the Au(111) spots are getting weaker. The ring-shaped pattern indicates the presence of 360° rotational domains with only weak favouritism towards the Au(111) direction. The XPS measurements show a 1:2 Co to Cl ratio, no peak shift compared to KCl (Cl^{1-} states at $E_B = 199$ eV for $2p_{3/2}$ and $E_B = 201$ eV for $2p_{1/2}$)[7] and a constant peak shape, indicating regular, stoichiometric growth.

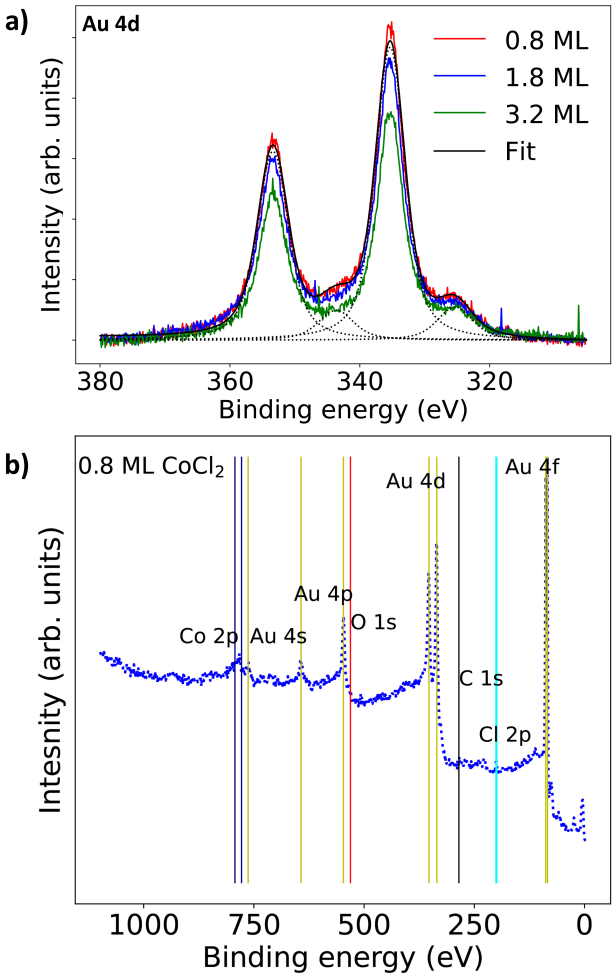

Figure 12: (a) Au 4d core-level XPS spectra for different coverages of $CoCl_2$ on Au(111). Here, the peak intensity is decreasing, due to the increased attenuation of the peak intensity of the substrate. (b) Example Survey Spectra of 0.4 ML $CoCl_2$ on Au(111) showing no impurities.

Table 1: Element-specific core-level peak positions and the corresponding full width half maximum (FWHM) including the respective error, which were obtained from the fitting routine (lmfit[3]). The fitting parameter for the Co peaks are listed without error values. This is due to the large amount of peaks partially overlapping, which are present in the same spectral region.

Coverage	Element-Peaks	FWHM (eV)	Peak position (eV)
0.8 ML	Cl 2p 3/2	1.82	199.59
	2p 1/2	1.91	201.20
0.8 ML	Au 4d 5/2	5.14	335.32
	4d 3/2	5.72	353.37
	Au 4d 5/2 (Al-anode)	6.48	325.63
	4d 3/2 (Al-anode)	6.48	343.54
0.8 ML	Co 2p 3/2	2.34	781.97
	Co 2p 1/2	2.34	798.00
	Co Sat. 2p 3/2	5.40	787.00
	Co Sat. 2p 1/2	5.40	803.41
	Au 4s	7.20	762.51
	Co 2p 3/2 (Al-anode)	4.25	772.50
	Co 2p 1/2 (Al-anode)	3.96	790.50
	Co 2p 3/2 sat (Al-anode)	5.40	777.00
	Co 2p 1/2 sat (Al-anode)	3.96	794.00
1.8 ML	Cl 2p 3/2	2.03	199.53
	2p 1/2	2.07	201.22
1.8 ML	Au 4d 5/2	5.10±0.02	335.35±0.01
	4d 3/2	5.73	353.40
	Au 4d 5/2 (Al-anode)	6.48	325.67
	4d 3/2 (Al-anode)	6.48	343.11
1.8 ML	Co 2p 3/2	2.34	781.92
	Co 2p 1/2	2.34	798.00
	Co Sat. 2p 3/2	6.30	787.50
	Co Sat. 2p 1/2	6.29	803.40
	Au 4s	8.28	762.73
	Co 2p 3/2 (Al-anode)	3.19	771.50
	Co 2p 1/2 (Al-anode)	3.96	788.95
	Co 2p 3/2 sat (Al-anode)	5.40	777.00
	Co 2p 1/2 sat (Al-anode)	3.96	794,23

Coverage	Element-Peaks	FWHM (eV)	Peak position (eV)
3.2 ML	Cl 2p 3/2	1.76	199.68
	2p 1/2	1.72	201.27
3.2 ML	Au 4d 5/2	5.02	335.30
	4d 3/2	5.49	353.36
	Au 4d 5/2 (Al-anode)	6.48	325.64
	4d 3/2 (Al-anode)	6.48	343.56
3.2 ML	Co 2p 3/2	2.34	782.06
	Co 2p 1/2	2.34	798.00
	Co Sat. 2p 3/2	6.30	787.50
	Co Sat. 2p 1/2	6.48	803.36
	Au 4s	8.28	761.00
	Co 2p 3/2 (Al-anode)	3.17	771.50
	Co 2p 1/2 (Al-anode)	3.96	788.95
	Co 2p 3/2 sat (Al-anode)	5.40	777.00
	Co 2p 1/2 sat (Al-anode)	3.96	794.00

Coverage Calibration XAS

The whiteline image of $CoCl_2$ can be used to calibrate the coverage on Au(111). Therefore, a comparison between the observed peak intensity of $FeBr_2$ on Au(111) is used.[4] Since at low temperature the Au background in the energy regime of Fe and Co behaves nearly identical,[8] the same calibration can be used. In the case of $FeBr_2$ on Au(111) a 0.7 ML sample had a average peak height of 14%. However, since the calculation is already based on a coverage with an error we need to assume a coverage error of ≈ 0.5 ML for all XAS measurements. The coverages for the XAS measurements are thus only a rough approximation.

Sum-rule analysis of the XMCD data

The effective spin magnetic moment and orbital magnetic moment values were calculated from the areas of the L_3 and L_2 peaks of the XMCD spectra from the sum rules:[9–11]

$$m_{s,eff} = -\frac{A_{L_3} - 2 \cdot A_{L_2}}{A_{\text{Average}}} \cdot N_h \cdot \frac{1}{\sigma} \tag{1}$$

$$m_l = -\frac{2}{3} \cdot \frac{A_{L_3} + \cdot A_{L_2}}{A_{\text{Average}}} \cdot N_h \cdot \frac{1}{\sigma} \tag{2}$$

, where N_h is the number of holes (= 3 for Co^{2+} in $CoCl_2$), A_{L_3}, A_{L_2} and A_{Average} are the areas of the XMCD L_3 and L_2 region and the total area of the isotropic XAS. σ represents the degree of circular polarization, which is beamline-dependent. The $m_{s,eff}$ value calculated via Equation (1) is the effective spin magnetic moment, result of the sum of the actual spin moment plus the magnetic dipole term, $\frac{7}{2}T_Z$. The background correction was performed using an asymmetrically reweighted penalized least- squares smoothing.[12] All calculated magnetic moments are shown in Table 2

Table 2: Samples measured by XMCD for different coverage (Θ) at T=2 K and B=6 T. The magnetic moments (μ) are calculated for the in-plane and out of plane measurements. The total magnetic moment m_{tot} per Co atom is the sum of the effective spin moment $m_{\text{s eff}}$ and the orbital moment m_l. The measured moments are shown for NI and GI with an error of ±10%.

Θ (ML)	T (K)	μ (μ_B/Co at)					
		NI			GI		
		$m_{\text{s eff}}$	m_l	m_{tot}	$m_{\text{s eff}}$	m_l	m_{tot}
0.4	2	0.35	0.17	0.52	1.81	0.91	2.72
	8	-	-	-	1.76	0.89	2.65
	15	-	-	-	1.79	0.87	2.66
	25	-	-	-	1.34	0.76	2.10
1.1	2	0.35	0.17	0.52	1.60	0.81	2.41
	8	-	-	-	1.63	0.78	2.41
	15	-	-	-	1.62	0.76	2.38
	25	-	-	-	1.41	0.67	2.08
1.4	2	0.36	0.18	0.54	1.60	0.83	2.43
	8	-	-	-	1.53	0.79	2.32
	15	-	-	-	1.52	0.77	2.29
	25	-	-	-	1.35	0.70	2.05

Magnetisation Curves and Arrott Plots

Figures 13 show the magnetization curves and the Arrott plots. The linear part of the Arrott plots is fitted by linear regression yielding intersects with the x-axis that can be used to get a rough estimation of the transition temperature.

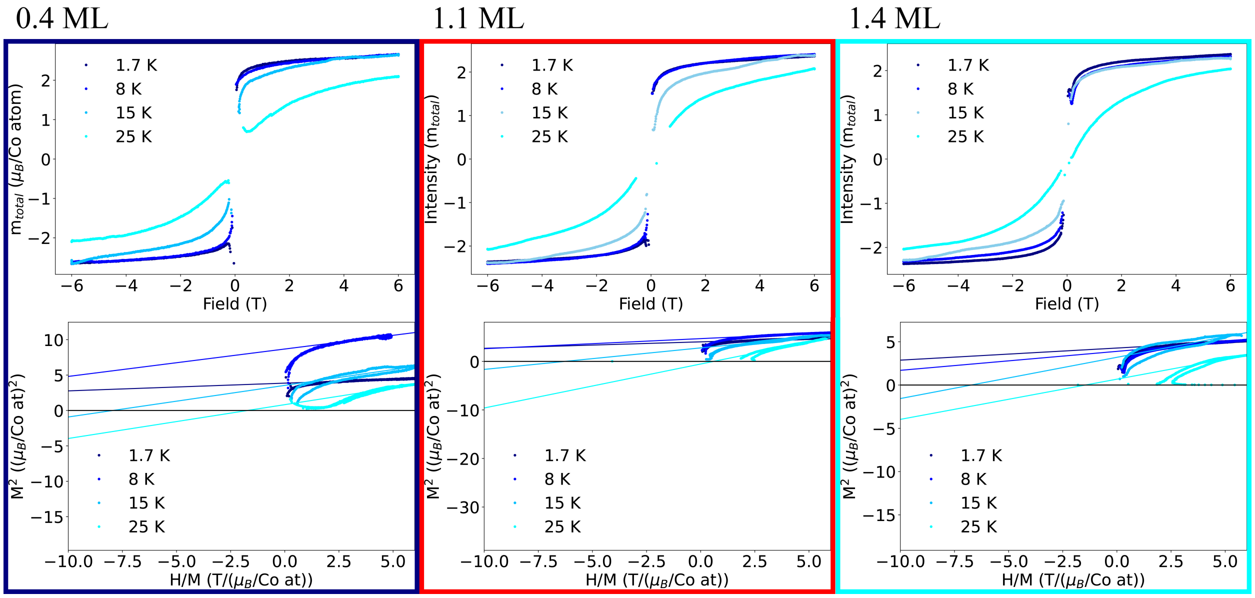

Figure 13: Temperature dependent magnetization curves of 0.4, 1.1, and 1.4 ML of $CoCl_2$ on Au(111) with respectively calculated Arrott plot. The magnetization curves are scaled to the expectation value of the total magnetic moment at 6 T. The intersect of the fitt of the linear part of the Arrott plot with the x-axis, plotted as a function of the measurement temperature can be used to determine the transition temperature.

The magnetocrystalline anisotropy energy E_{ani} is a measure of the energy required to rotate the magnetization from the easy axis to the hard axis. It can be estimated from the anisotropy field H_{ani} and the difference in magnetic moments $\Delta\mu$ using the following equations:

$$E = \Delta\mu \cdot H_{\text{ani}} \tag{3}$$

$$\Delta\mu = \mu_\perp - \mu_\| \tag{4}$$

where $\Delta\mu$ is the difference in magnetic moment between the hard axis (perpendicular direction) and the easy axis (parallel direction), $\mu_{\perp}$ is the magnetic moment measured at normal incidence (out-of-plane), $\mu_{\parallel}$ is the magnetic moment measured at grazing incidence (in-plane) and H_{ani} is the anisotropy field, i.e., the magnetic field required to align the magnetization along the hard axis. This quantity is calculated by extrapolating the in plane and out of plane magnetization curves until they intersect. E_{ani} is the resulting magnetic anisotropy energy.

References

(1) Materials Project. 2024-07-02; http://www.materialsproject.org.

(2) Schouteden, K.; Lievens, P.; Van Haesendonck, C. Fourier-transform scanning tunneling microscopy investigation of the energy versus wave vector dispersion of electrons at the Au(111) surface. Physical Review B **2009**, 79, 195409.

(3) Newville, M.; Stensitzki, T.; Allen, D. B.; Ingargiola, A. LMFIT: Non-Linear Least-Square Minimization and Curve-Fitting for Python. 2014; https://zenodo.org/record/11813.

(4) Hadjadj, S. E. et al. Epitaxial Monolayers of the Magnetic 2D Semiconductor $FeBr_2$ Grown on Au(111). Chemistry of Materials **2023**, 35, 9847–9856.

(5) Grosvenor, A. P.; Kobe, B. A.; Biesinger, M. C.; McIntyre, N. S. Investigation of multiplet splitting of Fe 2p XPS spectra and bonding in iron compounds. Surface and Interface Analysis **2004**, 36, 1564–1574.

(6) Bagus, P. S.; Nelin, C. J.; Al-Salik, Y.; Ilton, E. S.; Idriss, H. Multiplet splitting for the XPS of heavy elements: Dependence on oxidation state. Surface Science **2016**, 643, 142–149.

(7) Moulder, J. F., Chastain, J., Eds. Handbook of X-ray photoelectron spectroscopy: a reference book of standard spectra for identification and interpretation of XPS data, update ed.; Perkin-Elmer Corporation, 1992.

(8) Gullikson, E. Gold (Au) Z = 79, Energy = 1000.00 eV. (2024); https://henke.lbl.gov/cgi-bin/pert_cgi.pl.

(9) Kuch, W. X-ray Magnetic Circular Dichroism for Quantitative Element-Resolved Magnetic Microscopy. Physica Scripta **2004**, T109, 89.

(10) Chen, C. T.; Idzerda, Y. U.; Lin, H.-J.; Smith, N. V.; Meigs, G.; Chaban, E.; Ho, G. H.; Pellegrin, E.; Sette, F. Experimental Confirmation of the X-Ray Magnetic Circular Dichroism Sum Rules for Iron and Cobalt. Physical Review Letters **1995**, 75, 152–155.

(11) Carra, P.; Thole, B. T.; Altarelli, M.; Wang, X. X-ray circular dichroism and local magnetic fields. Physical Review Letters **1993**, 70, 694–697.

(12) Baek, S.-J.; Park, A.; Ahn, Y.-J.; Choo, J. Baseline correction using asymmetrically reweighted penalized least squares smoothing. The Analyst **2015**, 140, 250–257.
